# Assessing the impact of public health interventions on the transmission of pandemic H1N1 influenza a virus aboard a Peruvian navy ship

**DOI:** 10.1111/irv.12240

**Published:** 2014-02-10

**Authors:** Delphis M Vera, Ricardo A Hora, Anarina Murillo, Juan F Wong, Armando J Torre, David Wang, Darbi Boulay, Kathy Hancock, Jacqueline M Katz, Mariana Ramos, Luis Loayza, Jose Quispe, Erik J Reaves, Daniel G Bausch, Gerardo Chowell, Joel M Montgomery

**Affiliations:** a US Naval Medical Research Unit No. 6Lima, Peru; b Arizona State UniversityTempe, AZ, USA; c Centers for Disease Control and PreventionAtlanta, GA, USA; d Peruvian NavyLima, Peru; e Tulane UniversityNew Orleans, LA, USA; f National Institutes of HealthBethesda, MD, USA

**Keywords:** Disease outbreak, influenza, military personnel, Peru, ships, transmission

## Abstract

**Background:**

Limited data exist on transmission dynamics and effectiveness of control measures for influenza in confined settings.

**Objectives:**

To investigate the transmission dynamics of a 2009 pandemic H1N1 influenza A outbreak aboard a Peruvian Navy ship and quantify the effectiveness of the implemented control measures.

**Methods:**

We used surveillance data and a simple stochastic epidemic model to characterize and evaluate the effectiveness of control interventions implemented during an outbreak of 2009 pandemic H1N1 influenza A aboard a Peruvian Navy ship.

**Results:**

The serological attack rate for the outbreak was 49·1%, with younger cadets and low-ranking officers at greater risk of infection than older, higher-ranking officers. Our transmission model yielded a good fit to the daily time series of new influenza cases by date of symptom onset. We estimated a reduction of 54·4% in the reproduction number during the period of intense control interventions.

**Conclusion:**

Our results indicate that the patient isolation strategy and other control measures put in place during the outbreak reduced the infectiousness of isolated individuals by 86·7%. Our findings support that early implementation of control interventions can limit the spread of influenza epidemics in confined settings.

## Introduction

While several studies have analyzed the transmission dynamics and effectiveness of control interventions during the influenza A (H1N1)pdm09 virus pandemic at the community and regional levels,^1^–^4^ similar investigations focusing specifically on transmission in confined settings are limited. Historical evidence demonstrates that military populations, which often live and work in confined settings such as barracks or ships, are susceptible to respiratory disease outbreaks.^5^ Shipboard military populations may be especially susceptible due to crowded living conditions, stressful work environments, shared sanitation and ventilation systems, and the obligatory close proximity of large crews traveling together for prolonged periods of time,^6^ all of which provide an excellent environment for transmission of influenza and other respiratory viruses.^7^ Furthermore, shipboard personnel may acquire respiratory pathogens while in port and subsequently spread them to susceptible shipmates.^8^

A report of an H1N1pdm09 virus outbreak aboard a Peruvian Navy ship during an annual military strategic training exercise suggested that populations in confined military settings experience high rates of influenza during outbreaks and concluded that surveillance can be extremely important for timely disease detection and implementation of control measures to prevent the dissemination of respiratory pathogens.^9^ Timely detection of respiratory disease case clusters indicating epidemic potential may allow for triggering of specific public health investigations and targeted interventions and resource allocation to reduce transmission.^10^ Prompt implementation of enhanced infection control measures may be useful in controlling and preventing shipboard influenza outbreaks,^11^ and potentially applicable to other similar closed or semi-closed populations (e.g., schools, daycares, hospitals).

Disease transmission models are a useful tool to characterize the effect of timely detection and prompt implementation of interventions to mitigate the transmission of respiratory pathogens in confined settings. We used available data from the 2009 H1N1pdm09 influenza outbreak report aboard a Peruvian Navy ship to investigate the transmission dynamics and quantify the effectiveness of the implemented control measures. We used a stochastic epidemic model suitable for influenza transmission in confined settings that incorporates the effect of isolation strategies and changes in transmission rates associated with reactive control interventions during epidemics.

## Methods

### Data sources

Data from an onboard, ongoing surveillance system and from an outbreak investigation conducted upon the ship's return to Lima were used for analysis in this study.

#### Description of the outbreak response aboard the ship and the ongoing surveillance system

Surveillance data were obtained from a laboratory-based disease surveillance system aboard a Peruvian Navy ship on which an H1N1pdm09 outbreak occurred during June 2009. Details of the epidemiology of the outbreak have been previously described.^9^ Briefly, the ship, with a crew of 355 comprised mostly of young male cadets and low-ranking officers ages 18–35 years, departed Callao (in the Lima metropolitan area), Peru, on May 19 with stops in Ecuador, Costa Rica, California, Mexico, and Panama before returning to Peru on July 17. On June 25, a crew member reported to the infirmary with a 2-day history of influenza-like illness (ILI) and was subsequently confirmed to have H1N1pdm09 virus infection by real-time reverse transcriptase-polymerase chain reaction (rRT-PCR), which was suspected because the ship had recently made a 4-day port call to San Francisco, California, from June 20 to June 24, where the virus was known to be circulating.

The ship was part of a Peruvian Navy respiratory surveillance program in which health personnel were trained in respiratory disease surveillance and collection of nasopharyngeal swab specimens from persons with ILI. Personal protective equipment and training in proper respiratory hygiene were provided. Crew members were encouraged to seek medical attention through the ship's infirmary as soon as they developed signs or symptoms of respiratory illness (e.g., fever, cough, or sore throat). Once it was recognized that a shipboard outbreak was occurring, an active search of other crew members meeting the ILI case definition (oral temperature ≥38·5°C and cough or sore throat) was put in effect. Suspected ILI cases were then placed in isolation in the ship's infirmary under supportive care, given masks and hand sanitizers, and monitored daily for additional symptoms^9^,^12^ Patients were isolated for a minimum of 7 days (range 7–9 days) or until symptoms resolved. Only six persons received antiviral drugs, the majority >48 hours after symptom onset, and five of the six received only one dose. Only selected health personnel with adequate personal protective equipment and respiratory precautions were allowed to have contact with patients during their illness.

Daily case counts were kept and registered on designated case report forms. On July 5, due to the increasing number of ILI cases, an additional deck adjacent to the infirmary was made available for patient isolation. Instructions about cough etiquette (i.e. covering one's mouth when coughing) and hand washing were reinforced.

The original intent was for swab specimens to be tested on the ship for influenza virus by rRT-PCR.^13^ However, logistical problems prevented routine shipboard testing. Nevertheless, swabs were routinely collected and analyzed by influenza rapid test aboard the ship, and by rRT-PCR at NAMRU-6 after the ship's return.

#### Outbreak investigation upon the ship's return

The U.S. Naval Medical Research Unit No. 6 (NAMRU-6) in Lima, Peru, in collaboration with the Peruvian Navy and Ministry of Health, conducted an investigation immediately upon arrival of the ship to its home port. Because the outbreak investigation was part of a public health intervention, formal Institutional Review Board approval was not required.^9^ Blood samples were drawn from all available personnel on board for testing for H1N1pdm09-specific antibodies.^14^,^15^ Serum samples were tested by microneutralization (MN) and hemagglutination inhibition (HI) assays using an A/California/07/2009-like H1N1pdm09 virus. Individuals with serum antibody titers of ≥40 by MN and ≥20 by HI were considered seropositive. This combination of H1N1pdm09-specific antibody titers was shown to provide 90% sensitivity and 96% specificity for the detection of H1N1pdm09 infection using sera collected 15 or more days post-symptom onset from individuals <60 years of age and 92% specificity in those aged 60–79 years.^16^

### Attack rates

The clinical attack rate was obtained from the available data sources and was defined as the proportion of crew members who developed ILI and had laboratory confirmation of H1N1pdm09 virus infection by rRT-PCR testing. The serological attack rate was defined as the proportion of crew members who were seropositive for H1N1pdm09-specific antibodies.

### Transmission model

We compiled a time series of daily case counts by date of symptom onset for all personnel who presented to the infirmary with clinical symptoms matching the ILI case definition. Data regarding the implementation of control measures during the outbreak were obtained from the medical duty officer's daily activity log. We used a stochastic SEIR (susceptible-exposed-infectious-recovered) transmission model that is particularly suited to disease spread in small confined populations.^17^,^18^ This epidemic model accounts for the isolation of identified cases and time-dependent changes in transmission during different periods of intervention measures that were put in place during the outbreak on the ship.

Isolated individuals are assumed partially infectious, and the effectiveness of the isolation strategy is estimated from our model fit to H1N1pdm09 influenza case series data. The total crew size is assumed to be constant and initially completely susceptible to H1N1pdm09 virus infection. We also assumed a well-mixed crew population; that is, each individual had the same probability of having had contact with any other crew member given the small population setting. The goodness of fit of our transmission model was calculated using the chi-square goodness-of-fit test. We used available serological data as a way to validate our model-based inferences on the effect of mitigation strategies. Mathematical and computational modeling calculations were performed using MatLab (The Mathworks, Inc., Natick, MA, USA). Full descriptions of the transmission model and parameter estimations are provided in the accompanying Technical Appendix S1.

### Reproduction number and transmission potential

The effective reproduction number (R) accounts for changes in susceptibility and the effects of control interventions in the population as an outbreak unfolds and is thus useful in determining the effectiveness of control measures.^18^ The R for our model can be expressed as the sum of the contributions to infection from undetected sick crew members plus those persons who are placed in isolation and might be partially infectious. The formula for R is provided in the Technical Appendix S1.

### Statistical analysis

Univariate and bivariate analyses of results were performed using Stata 10 (StataCorp, College Station, TX, USA) employing chi-square (χ2), Fisher's exact, and Student t-tests as appropriate. *P* < 0·05 were regarded as statistically significant.

## Results

Of the 355 crew members, 23·9% (*n* = 85) presented to the infirmary meeting ILI criteria, of whom 91·8% (*n* = 78) tested positive for H1N1pdm09 virus by rRT-PCR (attack rate based on rRT-PCR-confirmed cases = 22·0%). Rapid tests were positive in 50 of the 85 ILI cases, yielding a sensitivity of 64% and specificity of 71%. Blood samples were collected from 79·7% (*n* = 283) of the crew, of whom 49% (*n* = 139) were antibody-positive, with younger cadets and low-ranking officers at higher risk than older, high-ranking officers (Table 1). Serological testing was performed on 57 of 58 individuals who tested positive by rRT-PCR. Eighty-four percent (*n* = 48) were antibody-positive. Of the nine seronegative persons, seven had sera collected <15 days post-symptom onset.

**Table 1:** Demographics, results of serologic testing, and attack rates by gender, rank, and age group during the outbreak of pandemic H1N1 influenza A on a Peruvian Navy Ship, June-July, 2009

Characteristic	No. samples drawn (%)	No. H1N1pdm09 specific antibody- positive (%)	Serological attack rate (%)	*P* value*
	*n* = 283	*n* = 139		
Gender				
Male	261 (92)	129 (46)	49	0·826
Female	22 (8)	10 (4)	46	
Rank				
Cadets**	140 (49)	89 (31)	64	<0·001
Low-ranking officers***	116 (41)	45 (16)	39	
High-ranking officers†	18 (6)	4 (1)	22	
Civilian	9 (3)	1 (0)	11	
Age group (years)				
18–25	161 (57)	98 (35)	61	<0·001
26–35	63 (22)	23 (8)	37	
36–45	29 (10)	9 (3)	31	
>46	30 (11)	9 (3)	30	

* Fisher's exact test.

** 2nd and 4th year trainee officers in the Peruvian Naval Academy.

*** Warrant officers, petty officers, and enlisted personnel.

† Junior, senior, and flag officers.

**Figure 1:**
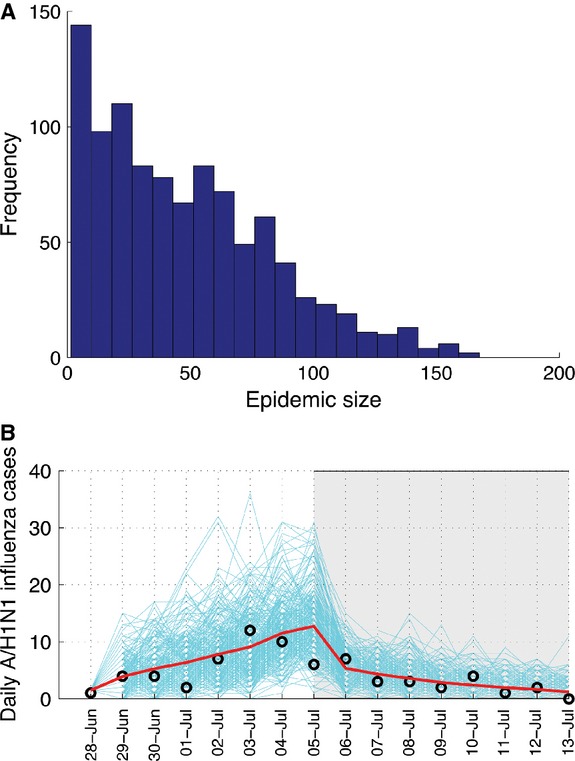
Influenza outbreak size distribution (Panel A) and corresponding fit of influenza virus transmission model with control interventions to the daily number of pandemic influenza A cases on the navy ship, June 28, to July 13, 2009 (Panel B). The final outbreak size histogram, which was obtained from 1000 stochastic epidemic simulations of the model calibrated to epidemic data, indicated epidemic extinction as the most likely outcome. The gray-shaded area indicates the period of patient isolation and other control measures implemented on July 5, 2009. Black circles represent the observed data. Blue lines are epidemic curves based on stochastic model realizations of the model best-fit. The red solid curve corresponds to the average of stochastic epidemic realizations.

**Figure 2:**
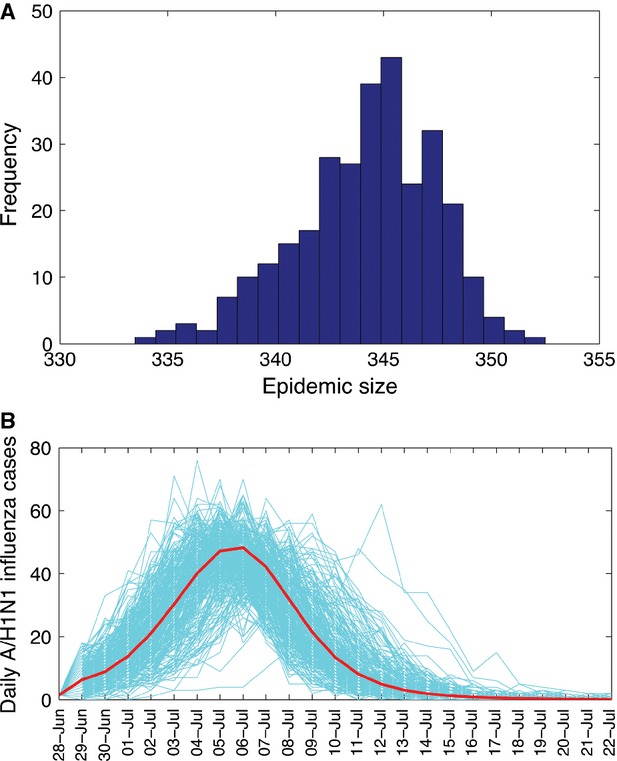
Influenza outbreak size distribution (Panel A) and stochastic epidemic curves in the absence of control interventions (Panel B). Final outbreak size histograms were obtained from 1000 stochastic epidemic simulations according to the epidemiology of influenza and reproduction number, *R* = 4·5. Epidemic curves were based on stochastic model realizations of influenza A (H1N1)pdm09 cases on the navy ship, June 28, to July 13, 2009. The red solid curve corresponds to the average of stochastic epidemic realizations.

Our transmission model yielded a good fit to the daily time series of new cases of H1N1pdm09 influenza by date of symptom onset (*P* = 0·46) (Figure 1). We identified a significant reduction in R during the period of intense control interventions. Specifically, we estimate that R decreased by 54·4% (95% CI 51·9–56·9), from 1·55 (95% CI 1·50–1·63) to 0·70 (95% CI 0·6–0·73) after the implementation of patient isolation and other control measures on July 5. Furthermore, we estimated that the isolation strategy implemented throughout the epidemic was associated with a reduction in the infectiousness of isolated individuals by 86·7% (95% CI 83–90). In the absence of the isolation strategy, we estimate *R* = 4·5, which corresponds to a mean clinical attack rate of 97% (Figure 2). Using our transmission model calibrated to H1N1pdm09 influenza case series data that included control measures, we estimated a probability of epidemic extinction of 68% in this small population setting using 1000 stochastic model realizations. That is, only 32% of stochastic model realizations yielded outbreaks. We also forecasted that additional 3- and 6-day delays in the implementation of control interventions would have yielded mean clinical attack rates of 32% and 39%, respectively. Similarly, our model indicates that a mean clinical attack rate of 19% would have been expected had intervention strategies started 3 days earlier than the actual start date.

## Discussion

The clinical attack rate found in the outbreak studied here was considerably higher than that reported for similar influenza A (H1N1) outbreaks in confined settings.^19^–^21^ The lower clinical attack rates seen in other settings can be attributed to several factors. In one previous influenza A (H1N1) outbreak, a ship was being pulled ashore for maintenance, with most crew members returning to their land-based residences daily, diminishing the effect of crowding and risk of transmission via respiratory droplets.^21^ Attack rates for influenza outbreaks aboard larger vessels, on which population density is reduced and which provide more space for patient isolation, may approach those seen in community settings;^4^ influenza A (H1N1) attack rates reported by Harwood *et al*. aboard two U.S. aircraft carriers, which are much larger than the ship involved in our study, were as low as 3%.^22^,^23^ Interestingly, the serological attack rate was more than double the clinical attack rate, which suggests that asymptomatic or mild illness occurred and/or that some sick crew members did not seek medical care at the ship's infirmary.

Although baseline serological data were not available from our crew members to demonstrate seroconversion, we believe that all antibody-positive results in our study reflect H1N1pdm09 virus infection occurring during the ship's voyage; although cases of H1N1pdm09 influenza had already been detected in Lima 1 week prior to the ship's departure, the first case aboard the ship was detected approximately 6 weeks after the ship left Peru, far beyond the 1- to 3-day incubation period for influenza.^24^ Furthermore, there was a clear temporal association of the ship's outbreak occurring immediately after the port call in San Francisco, where the H1N1pdm09 virus was in full force at the time.

Our relatively simple transmission model is particularly suited to disease spread in small confined populations and yielded a good fit to the case series data. Given the unique characteristics of the study population and transmission setting, higher clinical attack rates, up to 97%, could be expected under the “intervention-free” scenario. Our analysis showed that the clinical attack rate had a linear relationship with the delay in the implementation, with higher rates at later timing of implementation. Despite their potentially labor-intensive and expensive nature, our results indicate that early implementation of control measures reduces morbidity in confined settings.^25^ In particular, our results suggest that the timely implementation of patient isolation and other control measures were effective mitigation strategies. Moreover, our analysis of the available serological data is suggestive of the effectiveness of control measures in reducing transmission by more than 45%. Of note, we believe that it is highly unlikely that antiviral medications played a significant role in stemming the outbreak. We did not account for the effect of antiviral treatment on transmission in our model as this intervention likely had a negligible effect on transmission because only a few cases received antiviral treatment; treatment was started late in most cases (>48 hours from symptoms onset), and only one dose was administered.

The R of 1·55 calculated in our study is similar to those reported in other scenarios; an R between 1·4 and 3·1 was found in studies assessing the transmissibility of H1N1pdm09 virus in northern hemisphere community settings^2^,^26^ and 1·4 in a southern hemisphere setting.^27^ Studies assessing influenza virus transmissibility in confined settings during the 1918 pandemic have estimated R ranging from 2·1 to 7·5.^28^,^29^

We recognize numerous limitations in our study: (i) We assumed in our model that transmission among crew members occurred on the ship, but we cannot exclude the possibility of “spillover” transmission from the community while the ship was docked, and (ii) our relatively simple model yielded a good fit to the epidemic curve while assuming that mixing of crew members was well approximated by a random process, with an equal probability of contact between any two individuals. Nevertheless, there is probably higher contact between crew members of equal rank and similar duties than between ranks, which may account for the finding of higher probability of infection in a specific group (i.e., younger cadets and low-ranking officers).^9^ (iii) Similarly, we did not explicitly incorporate an age-specific mixing structure into our model given our relatively young and homogeneous study population. (iv) As numerous interventions were implemented essentially simultaneously (patient isolation and reinforcement of general hygiene and infection control measures, such as cough etiquette and frequent hand washing), it is not possible to determine the individual efficacy of any one of these measures. (v) Approximately 20% of the crew did not consent to blood draw, potentially skewing our results. However, in comparing the average age, sex, and military rank of participants and non-participants, the only significant difference was a higher number of males (*P* = 0·022, χ2) in the participants. Sex has not been shown to be a factor that directly affects influenza transmission, and thus, we do not feel that this is a biologically relevant difference. (vi) As described above, we cannot completely exclude the possibility that some crew members were infected with H1N1pdm09 virus prior to the ship's departure from Peru, because baseline serological data were not available from our crew members to demonstrate seroconversion.

Our findings suggest that reactive control interventions can effectively mitigate the impact of influenza outbreaks in confined settings when they are promptly implemented. Clinical and laboratory data provided a reliable index for the determination of changes in the R as a function of the implementation of interventions. Furthermore, many of the measures implemented on the ship may be applicable to other confined settings, such as child-care centers, nursing homes, and prisons. Future studies addressing other factors such as spatio-temporal distribution, changes in healthcare seeking behavior and social networking patterns during outbreaks, and adherence to mitigation interventions are needed to better understand the transmission dynamics of influenza in military confined settings.

## Addendum: author contributions

Dr Delphis M. Vera (delphis.vera@med.navy.mil), Dr Ricardo A. Hora (ricardo.hora@med.navy.mil), and Dr Gerardo Chowell (gchowell@asu.edu) were responsible for conception and design of the study; the acquisition, analysis and interpretation of data; drafting the article; critical revision of article, and final approval of article. Ms Anarina Murillo (anarina.murillo@asu.edu), Dr Juan F. Wong (juan.wong@med.navy.mil), and Dr Armando J. Torre (armando.torre@med.navy.mil): Contribution to the analysis and interpretation of data and critical revision of article. Dr Kathy Hancock (khancock07@comcast.net), Dr Jacqueline M. Katz (jmk9@cdc.gov): Laboratory procedures and critical revision of article. Dr David Wang (iuc9@cdc.gov) and Dr Darbi Boulay (DAranio@cdc.gov): Laboratory procedures. Dr Erik J. Reaves (erikreaves@gmail.com), Dr Mariana Ramos (mariana.ramos@med.navy.mil), Dr Luis Loayza (luis.loayza@med.navy.mil), and Mr. José Quispe (jose.quispe@med.navy.mil): Acquisition of data and critical revision of article. Dr Daniel G. Bausch (daniel.bausch@med.navy.mil) and Dr Joel M. Montgomery (jmontgomery@ke.cdc.gov): Drafting, critical revision, and final approval of article.

## Copyright Statement

Several authors of this work are employees of the US Government. This work was prepared as part of their official duties. Title 17 U.S.C 101 defines a U.S. Government work as a work prepared by a military service member or employee of the U.S. Government as part of that person's official duties.

## Supporting Information

**Appendix S1.** Influenza transmission model.

**Figure S1.** Schematic diagram of the epidemiological state progression modeled by our influenza transmission model.

## References

[1] Wu JT, Cowling BJ, Lau EH (2010). School closure and mitigation of pandemic (H1N1) 2009, Hong Kong. Emerg Infect Dis.

[2] Fraser C, Donnelly CA, Cauchemez S (2009). Pandemic potential of a strain of influenza A (H1N1): early findings. Science.

[3] Chowell G, Echevarria-Zuno S, Viboud C Characterizing the Epidemiology of the 2009 Influenza A/H1N1 Pandemic in Mexico. PLoS Med.

[4] Yu H, Cauchemez S, Donnelly CA (2012). Transmission dynamics, border entry screening, and school holidays during the 2009 influenza A (H1N1) pandemic, China. Emerg Infect Dis.

[5] Lim ML, Gabele SE, Wallace MR, Gray GC, Earhart KC (2004). Upper respiratory tract infections (URI). Mil Med.

[6] Kak V (2007). Infections in confined spaces: cruise ships, military barracks, and college dormitories. Infect Dis Clin North Am.

[7] Gray GC, Callahan JD, Hawksworth AW, Fisher CA, Gaydos JC (1999). Respiratory diseases among U.S. military personnel: countering emerging threats. Emerg Infect Dis.

[8] Ksiazek TG, Olson JG, Irving GS, Settle CS, White R, Petrusso R (1980). An influenza outbreak due to A/USSR/77-like (H1N1) virus aboard a US Navy ship. Am J Epidemiol.

[9] Centers for Disease C, Prevention (2010). Outbreak of 2009 pandemic influenza A (H1N1) on a Peruvian Navy ship - June-July 2009. MMWR Morb Mortal Wkly Rep.

[10] Greene SK, Kulldorff M, Huang J (2011). Timely detection of localized excess influenza activity in Northern California across patient care, prescription, and laboratory data. Stat Med.

[11] Khaokham CB, Selent M, Loustalot FV (2013). Seroepidemiologic investigation of an outbreak of pandemic influenza A H1N1 2009 aboard a US Navy Vessel-San Diego, 2009. Influenza Other Respi Viruses.

[12] Mott PJ, Sisk BW, Arbogast JW, Ferrazzano-Yaussy C, Bondi CA, Sheehan JJ (2007). Alcohol-based instant hand sanitizer use in military settings: a prospective cohort study of Army basic trainees. Mil Med.

[13] WHO http://www.who.int/csr/resources/publications/swineflu/CDCrealtimeRTPCRprotocol_20090428.pdf.

[14] WHO http://www.who.int/influenza/gisrs_laboratory/2010_12_06_serological_diagnosis_of_influenza_by_microneutralization_assay.pdf.

[15] Katz JM, Hancock K, Xu X (2011). Serologic assays for influenza surveillance, diagnosis and vaccine evaluation. Expert Rev Anti Infect Ther.

[16] Veguilla V, Hancock K, Schiffer J (2011). Sensitivity and specificity of serologic assays for detection of human infection with 2009 pandemic H1N1 virus in U.S. populations. J Clin Microbiol.

[17] Diekmann O, Heesterbeek J (2000). Mathematical Epidemiology of Infectious Diseases: Model-building, Analysis, and Interpretation.

[18] Anderson RM, May RM (1991). Infectious Diseases of Humans: Dynamics and Control.

[19] Tarabbo M, Lapa D, Castilletti C (2011). Retrospective investigation of an influenza A/H1N1pdm outbreak in an Italian military ship cruising in the Mediterranean Sea, May-September 2009. PLoS One.

[20] Dill CE, Favata MA (2009). Novel influenza A (H1N1) outbreak on board a US navy vessel. Disaster Med Public Health Prep.

[21] Crum-Cianflone NF, Blair PJ, Faix D (2009). Clinical and epidemiologic characteristics of an outbreak of novel H1N1 (swine origin) influenza A virus among United States military beneficiaries. Clin Infect Dis.

[22] Almond NB, Hollis EM, Von Thun AM (2009). Preliminary report: Outbreak of Novel H1N1 Influenza aboard USS Boxer, 29 June - 31 July 2009. Med Surv Mo Rep.

[23] Harwood JL, Lavan JT, Brand GJ (2013). Two aircraft carriers' perspectives: a comparative of control measures in shipboard H1N1 outbreaks. Disaster Med Public Health Prep.

[24] Ministerio de Salud del Peru (2009). Caracteristicas Epidemiologicas De La Nueva Influenza AH1N1 en el Peru.

[25] Ward KA, Armstrong P, McAnulty JM, Iwasenko JM, Dwyer DE (2010). Outbreaks of pandemic (H1N1) 2009 and seasonal influenza A (H3N2) on cruise ship. Emerg Infect Dis.

[26] Boelle PY, Bernillon P, Desenclos JC (2009). A preliminary estimation of the reproduction ratio for new influenza A(H1N1) from the outbreak in Mexico, March-April 2009. Euro Surveill.

[27] Munayco CV, Gomez J, Laguna-Torres VA (2009). Epidemiological and transmissibility analysis of influenza A(H1N1)v in a southern hemisphere setting: Peru. Euro Surveill.

[28] White LF, Pagano M (2008). Transmissibility of the influenza virus in the 1918 pandemic. PLoS One.

[29] Vynnycky E, Trindall A, Mangtani P (2007). Estimates of the reproduction numbers of Spanish influenza using morbidity data. Int J Epidemiol.

