# Supplementary material for: Assessing the impact of public health interventions on the transmission of pandemic H1N1 influenza a virus aboard a Peruvian navy ship
Source: Influenza Other Respir Viruses. 2014 Feb 10;8(3):353–9. doi: 10.1111/irv.12240 (PMC4181484; doi:10.1111/irv.12240)
Supplement: Supplementary file 1 — Appendix S1. Influenza transmission model. [file irv0008-0353-SD1.docx]

**TECHNICAL APPENDIX**

**Influenza transmission model**

We used a stochastic SEIR type (susceptible-exposed-infectious-recovered) transmission model^1, 2^ that takes into account the isolation of notified symptomatic individuals and time-dependent transmission rates during different periods of intervention measures put in place during the influenza A H1N1pdm09 virus outbreak on the Peruvian Navy ship described in the main text. Similar compartmental models have been previously developed to study the transmission dynamics of SARS and the 1918 influenza pandemic.^3, 4^

In our model (Figure S1), the population is divided in 5 categories: Susceptible (S), Exposed (E), Symptomatic and infectious (I), Isolated and partially infectious (J), and Recovered (P) individuals. Symptomatic infectious individuals are either isolated (category J) at the rate α following notification or recover without being diagnosed/reported at the rate γ (e.g., unreported mild infections). Isolated individuals are assumed partially infectious and the effectiveness of the isolation strategy is estimated from data as explained in the Parameter Estimation section below. The total crew size (N=355) is assumed constant and initially susceptible to infection with H1N1pdm09 virus. We also assume a well-mixed crew population. That is, each individual has the same probability of having contact with any other. This assumption was made, in part, due to the small population setting and the lack of statistical power to analyze the relatively small numbers of crew members comprising individual ranks or age groups.^5^ Lastly, we assumed that previous influenza vaccination had no effect on virus transmission, given previous data showing that influenza vaccines being used prior to the 2009 pandemic afforded little protection against H1N1pdm09 virus.^6, 7^

Susceptible crew members infected with the virus enter the latent period (category E) at the rate β(t) (I(t) + l J(t))/N where β(t) is the mean transmission rate per day and at time t and *l* is the relative infectiousness of isolated individuals. We assumed an initial transmission rate (modeled by parameter β_1_) that remained constant prior to the start of the intense public health measures implemented on July 5^th^ and changed to β_2_ during the intervention period (where β_2_ < β_1_ if interventions were effective). The fraction (I(t)+*l*J(t))/N is the probability of contacting an infectious individual out of the total population size N. Latent individuals progress to the infectious and symptomatic class at the rate $\kappa$ (1/$\kappa$ is the mean latent period). The mean infectious period is given by 1/γ. Recovered individuals are assumed protected for the duration of the epidemic. The deterministic model that describes the above transmission process is given by:

dS/dt = – β(t) S (I+ *l* J)/N

dE/dt = β(t) S (I+ *l* J)/N – $\kappa$ E

dI/dt = $\kappa$ E - γ I - α I

dJ/dt = α I - γ J

dP/dt = γ (I+J)

and the transmission rate is given by the following function:

$$\beta(t)=\left\{\begin{matrix} \beta_{1}\text{ t<t}_{\text{July 5th - intervention}} \\ \beta_{2}\text{ t>=t}_{\text{July 5th-intervention}} \end{matrix}\right.$$

where t_July_5th_intervention_ is the day of the start of intense public health measures on July 5^th^. The system of ordinary differential equations was solved numerically using Matlab (The Mathworks, Inc).

**Stochastic simulation of epidemics**

It is particularly important to consider stochastic rather than deterministic model simulations due to higher stochasticity associated with smaller populations such as those confined in military ships. For this purpose, we generated stochastic epidemic realizations of the model described above through a Poisson simulation approach^8^ based on our deterministic model described above. We also explored the outbreak size distribution as a function of the timing and intensity of reactive control interventions.

**The reproduction number**

The basic reproduction number (R_0_) is defined as the number of secondary cases generated by a primary infectious case during its entire period of infectiousness in a completely susceptible population and in the absence of control interventions.^9-12^ When R_0_ is greater than one, transmission can occur, potentially resulting in an epidemic. One of the goals of public health interventions is to reduce this quantity to a number less than one as soon as possible. A more practical quantity is the effective reproduction number, R, which accounts for changes in susceptibility in the population as an epidemic outbreak unfolds in the population and the effects of control interventions. Reproduction numbers are useful in determining the intensity of interventions that are needed to bring an epidemic under control.^2^

Once epidemiological parameters are estimated, the reproduction number can be estimated by plugging in the estimated parameter values into a formula of the reproduction number. A formula for the reproduction number of our model can be expressed as the sum of the contributions to infection from symptomatic and infectious individuals in the ship that are not yet notified (class I) and those persons that are isolated and are partially infectious (class J).

The contribution of the infectious and not yet notified individuals to the reproduction number is given by:

R ^symptomatic^ = (Transmission rate) × (Mean infectious period)

The contribution of the isolated and partially infectious individuals to the reproduction number is given by:

R ^isolated^ = (Fraction of symptomatic and infectious cases that are isolated) × (Transmission rate) × (Relative infectiousness of isolated individuals) x (Mean infectious period)

Hence, the overall reproduction number is then given by:

R = R ^symptomatic^ + R ^isolated^

**Parameter estimation**

The mean latent period was fixed to 1.5 days and the mean infectious period was bounded in the range 2-3 days according to the epidemiology of H1N1pdm09 virus.^13-15^ The average time from symptoms onset to notification or notification rate (α) was fixed to 1/1.6 days according to the empirical distribution of notification delays of reported cases with laboratory-confirmed H1N1pdm09 influenza on the ship. To quantify changes in the transmission rate and effectiveness of isolation strategies, a simple epidemic model was fitted to the H1N1pdm09 influenza case epidemic curve by dates of symptom onset by relying on the general approach of “trajectory matching,” where one searches for the combination of model parameters that produces an epidemic curve most statistically similar to the observed one.^9, 10^ Once epidemiological parameters are estimated, these are used to generate estimates of R using a formula derived from the transmission model.

The transmission rates (β_1_ and β_2_), the relative infectiousness of the isolated individuals (*l*), and the initial numbers of individuals in the exposed E(0) and infectious I(0) categories were estimated by least squares fitting of the model to the daily number of new H1N1pdm09 virus cases by dates of symptoms onset. Due to the short latent period characteristics of influenza,^11, 16^ we assumed E(0) = I(0); this simplification allowed us to estimate only four parameters from the time series of laboratory-confirmed H1N1pdm09 virus cases. The reproduction number was estimated using data comprising the epidemic period preceding the start of the intensification of control interventions on July 5^th^.

**Uncertainty analyses**

We estimated the uncertainty of the estimated model parameters via parametric bootstrap as in previous studies.^4, 12^ Briefly, we simulated 100 alternate realizations of the epidemic trajectory, by perturbation of the best-fit curve of daily number of new H1N1pdm09 cases. We added to the best-fit curve a simulated error structure computed using the increment in the “true” number of cases from day j to day j+1 as the Poisson mean for the number of new cases observed in the j to j+1 interval. The 95% bootstrap-based confidence intervals for the reproduction number should be interpreted as containing 95% of estimates if the analysis was repeated with the same model assumptions and if observational error was the only source of noise.

**REFERENCES**

1. Diekmann O, Heesterbeek J. Mathematical Epidemiology of Infectious Diseases: Model-building, Analysis, and Interpretation. West Sussex: Wiley; 2000.

2. Anderson RM, RM M. Infectious diseases of humans: Dynamics and Control. Oxford: Oxford University Press; 1991.

3. Chowell G, Fenimore PW, Castillo-Garsow MA, Castillo-Chavez C. SARS outbreaks in Ontario, Hong Kong and Singapore: the role of diagnosis and isolation as a control mechanism. J Theor Biol. 2003 Sep 7;224(1):1-8.

4. Chowell G, Ammon CE, Hengartner NW, Hyman JM. Estimation of the reproductive number of the Spanish flu epidemic in Geneva, Switzerland. Vaccine. 2006 Nov 10;24(44-46):6747-50.

5. Boone JD, McGwire KC, Otteson EW, DeBaca RS, Kuhn EA, St Jeor SC. Infection dynamics of Sin Nombre virus after a widespread decline in host populations. Am J Trop Med Hyg. 2002 Sep;67(3):310-8.

6. Hancock K, Veguilla V, Lu X, Zhong W, Butler EN, Sun H, et al. Cross-reactive antibody responses to the 2009 pandemic H1N1 influenza virus. N Engl J Med. 2009 Nov 12;361(20):1945-52.

7. Effectiveness of 2008-09 trivalent influenza vaccine against 2009 pandemic influenza A (H1N1) - United States, May-June 2009. MMWR Morb Mortal Wkly Rep. 2009 Nov 13;58(44):1241-5.

8. Gustafsson L, Sternad M. Bringing consistency to simulation of population models--Poisson simulation as a bridge between micro and macro simulation. Math Biosci. 2007 Oct;209(2):361-85.

9. Chowell G, Ammon CE, Hengartner NW, Hyman JM. Estimating the reproduction number from the initial phase of the Spanish flu pandemic waves in Geneva, Switzerland. Math Biosci Eng. 2007 Jul;4(3):457-70.

10. Chowell G, Nishiura H, Bettencourt LM. Comparative estimation of the reproduction number for pandemic influenza from daily case notification data. J R Soc Interface. 2007 Feb 22;4(12):155-66.

11. Longini IM, Jr., Halloran ME, Nizam A, Yang Y. Containing pandemic influenza with antiviral agents. Am J Epidemiol. 2004 Apr 1;159(7):623-33.

12. Chowell G, Miller MA, Viboud C. Seasonal influenza in the United States, France, and Australia: transmission and prospects for control. Epidemiol Infect. 2008 Jun;136(6):852-64.

13. Fraser C, Donnelly CA, Cauchemez S, Hanage WP, Van Kerkhove MD, Hollingsworth TD, et al. Pandemic potential of a strain of influenza A (H1N1): early findings. Science. 2009 Jun 19;324(5934):1557-61.

14. Yang Y, Sugimoto JD, Halloran ME, Basta NE, Chao DL, Matrajt L, et al. The transmissibility and control of pandemic influenza A (H1N1) virus. Science. 2009 Oct 30;326(5953):729-33.

15. Cauchemez S, Donnelly CA, Reed C, Ghani AC, Fraser C, Kent CK, et al. Household transmission of 2009 pandemic influenza A (H1N1) virus in the United States. N Engl J Med. 2009 Dec 31;361(27):2619-27.

16. Ferguson NM, Cummings DA, Cauchemez S, Fraser C, Riley S, Meeyai A, et al. Strategies for containing an emerging influenza pandemic in Southeast Asia. Nature. 2005 Sep 8;437(7056):209-14.

**FIGURE LEGENDS**

**Figure S1.** Schematic diagram of the epidemiological state progression modeled by our influenza transmission model.
