# Supplementary figures and images for: Assessing the impact of public health interventions on the transmission of pandemic H1N1 influenza a virus aboard a Peruvian navy ship
Source: Influenza Other Respir Viruses. 2014 Feb 10;8(3):353–9. doi: 10.1111/irv.12240 (PMC4181484; doi:10.1111/irv.12240)

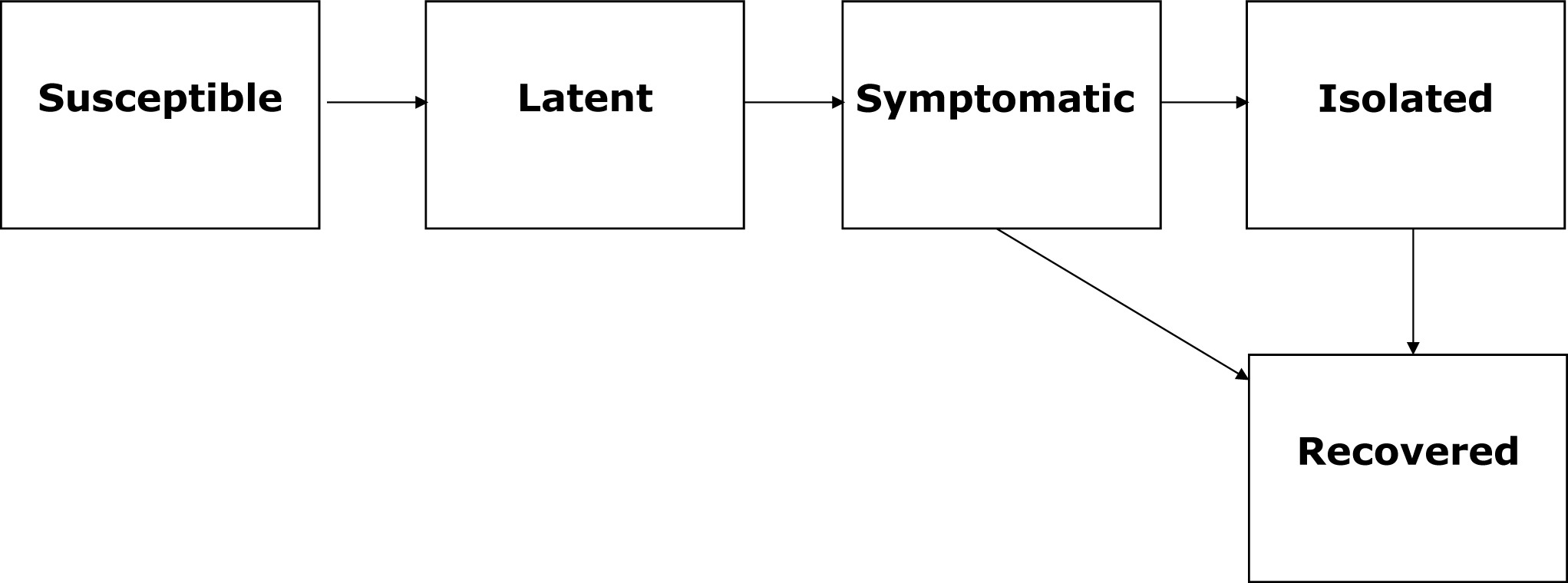

Supplement: Supplementary file 2 — Figure S1. Schematic diagram of the epidemiological state progression modeled by our influenza transmission model. [file irv0008-0353-SD2.tif]
